# Honeybee economics: optimisation of foraging in a variable world

**DOI:** 10.1038/srep28339

**Published:** 2016-06-20

**Authors:** Anton Stabentheiner, Helmut Kovac

**Affiliations:** 1 Institute of Zoology, University of Graz, Universitätsplatz 2, 8010 Graz, Austria

## Abstract

In honeybees fast and efficient exploitation of nectar and pollen sources is achieved by persistent endothermy throughout the foraging cycle, which means extremely high energy costs. The need for food promotes maximisation of the intake rate, and the high costs call for energetic optimisation. Experiments on how honeybees resolve this conflict have to consider that foraging takes place in a variable environment concerning microclimate and food quality and availability. Here we report, in simultaneous measurements of energy costs, gains, and intake rate and efficiency, how honeybee foragers manage this challenge in their highly variable environment. If possible, during unlimited sucrose flow, they follow an ‘investment-guided’ (‘time is honey’) economic strategy promising increased returns. They maximise net intake rate by investing both own heat production and solar heat to increase body temperature to a level which guarantees a high suction velocity. They switch to an ‘economizing’ (‘save the honey’) optimisation of energetic efficiency if the intake rate is restricted by the food source when an increased body temperature would not guarantee a high intake rate. With this flexible and graded change between economic strategies honeybees can do both maximise colony intake rate *and* optimise foraging efficiency in reaction to environmental variation.

Economic principles play an important role not only in human but also in animal communities^1^,^2^,^3^,^4^,^5^. The application of economic principles by animals implies finding a positive balance between energetic costs and gains, or between investment and returns^2^,^3^. Among the insects, foragers like honeybees are of special interest because they combine high energetic costs with high gains in a widely and wildly fluctuating environment. The high costs result from endothermy kept up throughout the foraging cycle (Fig. 1)^6^,^7^,^8^,^9^,^10^. High energetic gains are possible because they not only forage pollen for protein supply but also nectar and honeydew containing considerable amounts of sugars. A huge need for often only temporally accessible food for brood rearing and overwintering promotes maximisation of the intake rate^3^. Endothermy makes immediate flight possible even with heavy loads of up to the bees’ own body weight, and this way favours fast exploitation of resources^10^. Due to their small size, however, bees have to cope with an enormous heat loss^1^,^11^,^12^ and therefore high costs of thermoregulation^13^,^14^,^15^,^16^. These high costs call for energetic optimisation. Honeybees, however, do not forage in a constant but in a variable world^6^,^7^,^10^,^17^,^18^ where environmental variation not only refers to microclimatic conditions like temperature and insolation but also to variation in food quality and availability (e.g. sugar content and amount of nectar per flower).

From an economic point of view, two main economic principles are suggested to govern honeybee foraging behaviour: Following an ‘investment-guided’ (or ‘investing’) strategy^16^ means investing additional resources even under seemingly unfavourable conditions because this promises increased returns. Increased returns might be realized through an increase of the intake rate, e.g. gathered energy (amount of sugar solution) or pollen per time interval. An ‘economizing’ strategy^16^, by contrast, would reduce energetic investment and thus costs whenever possible. This could be realized by reducing the bees’ own heat production with increasing ambient temperature (T_a_) because heat loss decreases accordingly if body temperature remains constant, and by using external heat from the sun to save energy for thermoregulation. In other words, energetic efficiency should be in the fore in this case. Theoretical considerations have claimed both strategies to occur in social insect foraging^3^. Experimental research on this topic, however, has been stuck for a couple of years now, in part due to considerable variation of environmental and experimental conditions which makes energetic estimations imprecise. Physiological parameters and constraints^4^,^10^ have often been neglected in theoretical considerations. To elucidate the general economic principles acting during honeybee foraging we therefore used simultaneous measurements of CO_2_ production (to calculate energy costs), energy gain (via the gathered amount of sugar at an artificial flower), body temperature and microclimatic conditions, to approach an empirical decision of how honeybee foragers master the challenge of balancing food intake rate and gains with energetic costs in reaction to their highly variable environment.

## Results and Discussion

### High investment for high quality resources

**Figure 1:**
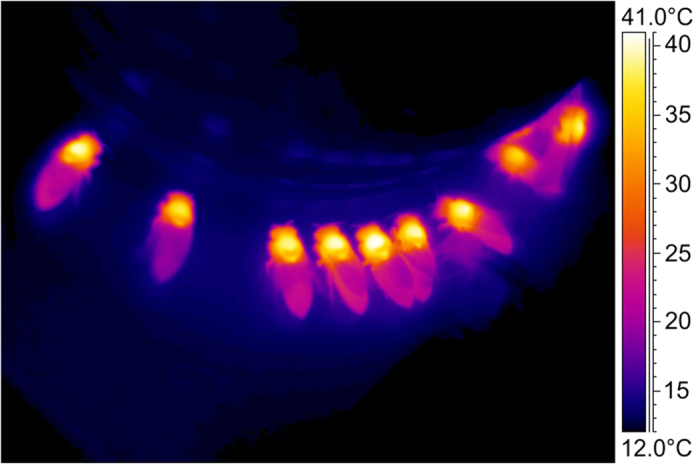
Infrared thermogram of honeybees (*Apis mellifera carnica*) foraging sucrose solution. Note heated thoraxes resulting from intense endothermy with activated flight muscles. Ambient air temperature = 12 °C.

**Figure 2:**
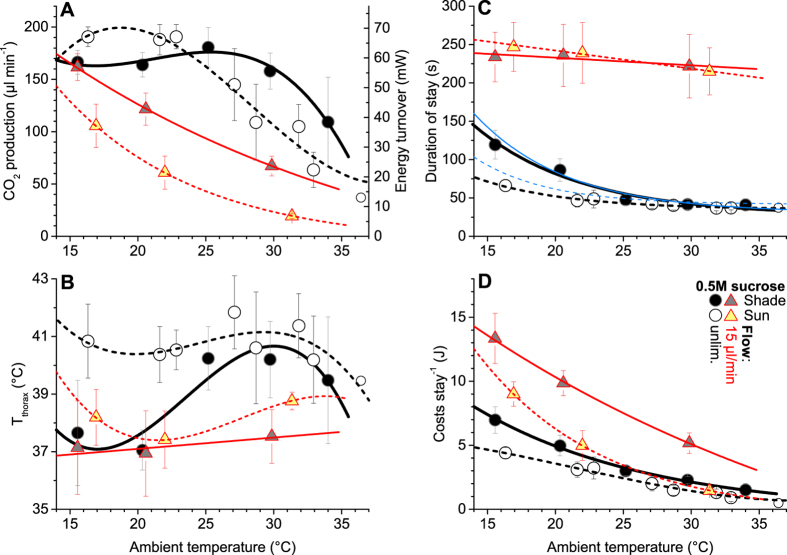
Energetics and thermoregulation of sucrose foraging honeybees. (**A**) CO_2_ production rate and energy turnover. (**B**) Thorax surface temperature, for head and abdomen see Supplementary Fig. S1. (**C**) Duration of stay. Blue thin lines: 1.5 M feeding at unlimited flow, solid = shade, dashed = sun, from^16^. (**D**) Energy costs per stay. (**A–D**) 22 individuals of *Apis mellifera carnica* foraging 0.5 M sucrose provided in unlimited (unlim.) flow or at a rate of 15 μl/min, in shade (solid lines) or in sunshine (dashed lines), 504 visits, for legend see (**D**). Symbols represent means with SD of individual stays shown in Supplementary Fig. S3; for radiation values see Supplementary Fig. S2, and for regression functions and statistics see Supplementary Table S1.

**Figure 3:**
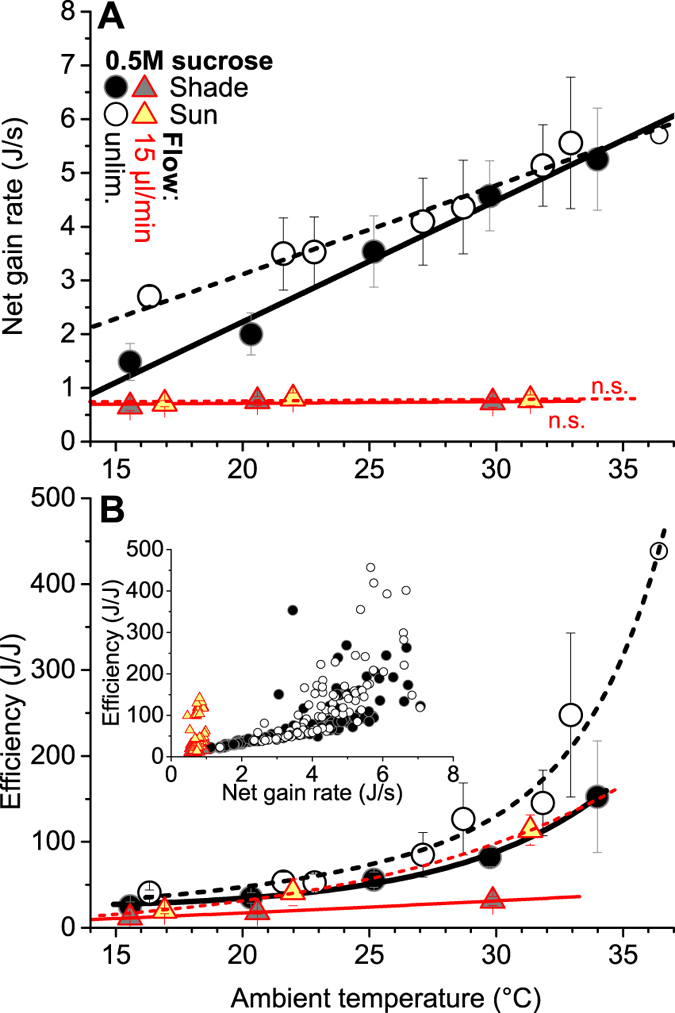
Net energy gain rate and foraging efficiency of honeybees. (**A**) Net energy gain rate per stay (gain-costs/second, in J/s), regressions for unlimited (unlim.) flow different in slope and intercept (P < 0.0005, ANOVA). (**B**) Foraging energy efficiency during the stays at the feeder (gain-costs/costs, in J/J), correlations different in slope and intercept (P < 0.01) except unlimited flow in shade and 15 μl/min in sun (ANOVA); insert, relationship between efficiency and net gain rate (individual stays). (**A,B**) Symbols represent means with SD of individual stays. Main graphs: all relationships significant at P < 0.0001 except n.s. in (**A**), for regression functions and statistics see Supplementary Table S1. Insert: P ≪ 0.0001 for unlimited flow in shade and sunshine; P < 0.01 in shade and n.s. in sunshine for 15 μl min^−1^ flow. For individual stays see Supplementary Fig. S4.

It turned out that, while the relationships between energy turnover, body temperature regulation and the effect of environmental parameters on both seem complex in detail (Fig. 2A,B), the basic economic rules governing honeybee thermoregulation and energetics are rather simple, resembling principles of human economics^2^. The foragers remained endothermic during the whole foraging stays (compare Fig. 1) but the level of thermoregulation differed considerably in dependence on environmental and feeding conditions, mean thorax temperatures ranging from ~37 °C to 42 °C (Fig. 2B). It was a surprising finding that during unlimited sucrose flow (0.5 mol/l) bees foraging in shade kept the own heat production rather high and constant (~58–62 mW on average) up to an ambient temperature (T_a_) of ~29 °C (Fig. 2A) despite a decreasing difference of the body surface temperature to T_a_ (see Supplementary Fig. S1). Only at the highest T_a_ (>29 °C) they reduced the own energetic effort in part. This range of constant heat production may be extended to even higher T_a_ at more profitable food sources (e.g. 1.5 mol/l sucrose)^16^. The endogenous heat was invested to increase the thorax temperature from about 37 °C at low T_a_ to ~39.5–40.5 °C at high T_a_ (P < 0.0001, t = 13.4428, df = 215) (Fig. 2B). With increasing T_a_ also the temperatures of head and abdomen increased (see Supplementary Fig. S1). It was mainly this increase of body temperature which enabled the bees to ingest the sucrose solution faster (reduce the duration of stay, Fig. 2C) and this way reduce energetic costs per visit at higher T_a_ (Fig. 2D). In an economic sense, the bees acted ‘investment-guided’ under these profitable conditions, investing energy (instead of saving it) in a wide range of T_a_ to speed up foraging and thus increase intake (ingestion) rate with increasing T_a_ (Fig. 3A).

### Differential use of solar heat gain

In sunshine, during unlimited sucrose flow the foragers even increased the own heat production at low T_a_ (<25 °C) instead of using it to reduce energy turnover (P < 0.0001, t = 9.039, df = 113) (Fig. 2A; for radiation values see Supplementary Fig. S2)! As a consequence they were able to increase the thorax temperature by ~2–3 °C (P < 0.0001, t = 14.775, df = 128) (Fig. 2B), which allowed them to considerably speed up food ingestion (Fig. 2C) and increase net energy intake rate at low T_a_ (Fig. 3A). At higher T_a_ (>25 °C), by contrast, the bees used solar heat to save own heat production (Fig. 2A). They could do this because their body temperature was already high enough (Fig. 2B) to guarantee a high ingestion rate (Figs 2C and 3A)^10^. The general validity of these regulatory principles and of the change between them is emphasized by similar findings in Vespine wasps foraging sucrose^19^.

### Limited sucrose flow promotes switch to economizing behaviour

It has to be considered, however, that in nature the nectar uptake rate is mostly limited by the nectar production of the flowers and not by the bees’ ingestion capacity. Therefore we limited the sucrose flow to 15 μl/min. At the lowest T_a_ the bees’ heat production rate in shade did not differ from that during unlimited flow (n.s., t = 1.5088, df = 56) (Fig. 2A) though they had to wait considerably longer to fill their crop (Fig. 2C). We suggest that, because of the high heat loss^1^,^11^, the bees had no choice but to invest a considerable amount of energy to keep their thorax temperature at a level high enough for proper take-off (~ 37 °C). By keeping this level of thermoregulation (Fig. 2B) they were able to reduce the own heat production rate considerably with increasing T_a_, following an ‘economizing’ strategy throughout their range of foraging T_a_ (Fig. 2A). Solar heat was only to a small extent invested to increase the thorax temperature (Fig. 2B). Most of it was used to save much of the own energy investment (Fig. 2A,D). The bees followed an ‘economizing’ strategy throughout the whole investigated range of T_a_ in this case.

### Bees optimise both intake rate and efficiency

A basic question in honeybee foraging optimisation is whether they primarily maximise the intake rate or the energetic efficiency^3^,^20^,^21^,^22^,^23^. Ydenberg *et al*.^3^ suggested that foragers may be ‘energy limited’ at one time, meaning that they behave as time minimisers (= rate maximisers; resembling our unlimited flow condition), or they may be ‘time limited’ at other times, meaning that they behave as energy minimisers (= efficiency maximisers; resembling our limited flow condition). With our simultaneous measurements of thermoregulation, energetic costs and energy gains from food we provide evidence that at a food source bees do both, maximise intake rate whenever possible but nevertheless optimise energetic efficiency if necessary and of benefit^10^,^16^.

In the first place, they always try to maximise the intake rate, following a ‘time is honey’^2^ rule. In a more natural situation this is possible during water gathering^10^,^24^, in some cases during honeydew collection if large droplets are available, or probably during honey robbery from foreign colonies. To achieve a high intake rate, the main parameter to be optimised is body temperature^10^.

If the intake rate cannot be increased because of limited food availability, energetic optimisation comes to the fore, the bees now following more a ‘save the honey’ rule. In this case it is important to keep the body temperature high enough for proper take-off (Fig. 2B) but as low as possible to minimise heat loss and energetic investment^1^,^11^,^12^. This means that the need to optimise body temperature prevents a further reduction of investment. The importance of temperature for the bees’ lift-off capacity^25^ is emphasized by increased flight energy requirements with increasing load^26^ and by their tendency to have a higher thorax temperature when leaving a food or water source fully loaded than when empty upon arrival^10^,^17^. Our limited-flow condition resembles the natural situation on composite plants like dandelion (*Taraxacum* sp.), sunflower (*Helianthus* sp.) or thistle (*Cirsium* sp.), with relatively long residence times on one inflorescence^27^,^28^ and few flights between flowers. Longer and more frequent flights between flowers on plants like apricot (*Prunus* sp.) or raspberry (*Rubus* sp.), however, do not necessarily mean higher energetic costs. Metabolism in flight is similar to and sometimes even lower^14^,^15^,^26^ than at our artificial flowers (Fig. 2A)^16^,^29^. In a natural situation on flowers, therefore, efficiency optimisation will be the more important strategy^21^.

### Foraging efficiency strongly determined by environment

**Figure 4:**
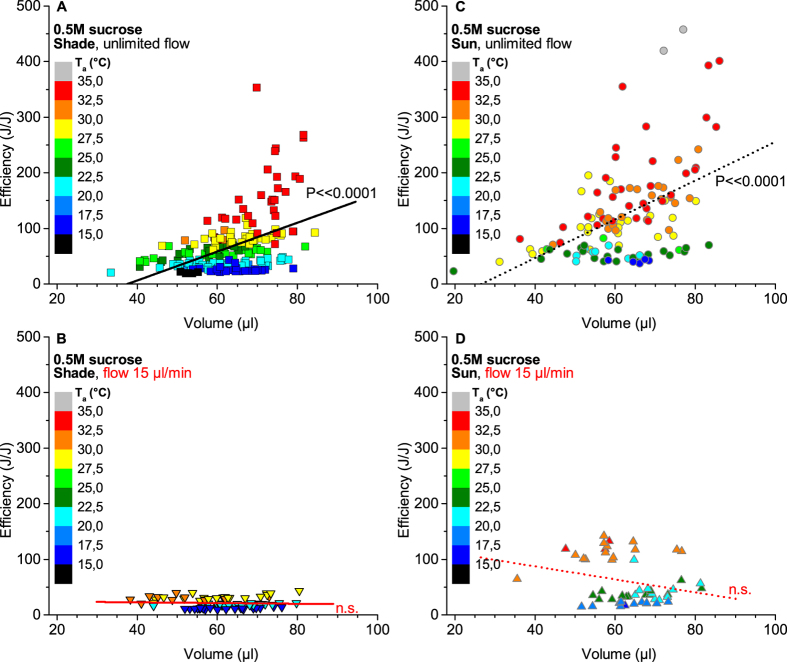
Net energy efficiency in relation to sucrose ingestion volume. (**A–D**) Different sucrose flow and radiation conditions. Colouring of values according to ranges of ambient air temperature (T_a_) as shown by colour scales. Symbols represent individual stays. (**B,D**) regressions not significantly different from zero (n.s.).

With our experimental approach we were not able to verify the hypothesis that honeybees maximise net energetic foraging efficiency (gain-costs/costs; in J/J)^23^ during their stay at a food source by not filling their crop^30^, similar to experiments with even lower flow rates and the bees flying between artificial flowers^31^. Either there was no effect, during limited sucrose flow, or efficiency even increased with the ingested volume during unlimited flow (Fig. 4). This is also valid if one compares the energetic efficiency for certain ranges of T_a_ only (see colour scales in Fig. 4). Efficiency turned out to be much more dependent on environmental conditions, increasing with ambient temperature especially strong during unlimited feeding and even more during foraging in sunshine (Fig. 3B). Earlier predictions from optimal foraging theory had suggested that central place foragers like honeybees optimise total net daily (energy) gain^3^. It had remained unclear, however, whether they achieve this by minimising time (i.e. maximising individual intake rate) or by minimising costs (i.e. maximising efficiency). One might argue that it may be impossible to simultaneously optimise these two seemingly contrasting criteria. However, our experiments with unlimited sucrose flow show that it is possible and that honeybees do it. At high ambient temperature (T_a_ > ~30 °C) the duration of stay in shade tends towards a minimum (Fig. 2C), and this way food intake rate is obviously maximised. Since a further rate increase seems not possible (at least not with the body temperature the bees regulate under these conditions) they can reduce the costs (Fig. 2D) at a similar energy gain (see Supplementary Fig. S2), which increases efficiency (Fig. 3B). With external heat gain from the sun which the bees use to decrease the duration of stay (Fig. 2C) the maximum intake rate is approximated at lower T_a_ (>~23 °C) and this way reduction of the own energetic investment is already possible at this lower T_a_ (>~23 °C; Fig. 2A). Our experiments therefore provide direct empirical evidence that honeybees can optimise foraging not only by ‘switching’ between both strategies in reaction to environmental conditions but by a graded transition between both criteria, realized by regulating the key parameter body temperature up or down to achieve an optimal balance between intake rate and efficiency (or costs). Similar findings in Vespine wasps foraging at unlimited sucrose flow^19^ show that this dual optimisation is not restricted to honeybees but very likely represents a general principle in heterothermic insects with similar foraging practice.

The question arises why the foragers did not increase body temperature further to achieve an even higher intake rate. A comparison with earlier measurements during unlimited foraging of higher concentrated 1.5 M sucrose shows that they can do so^16^. However, though under those conditions they in part had regulated the thorax temperatures at a higher level (at higher costs) the duration of stay was nearly identical in shade and even somewhat higher in sunshine (see thin blue lines in Fig. 2C). We suggest that this is due to the exponential increase of sucrose viscosity with concentration^32^. The decrease of viscosity with temperature^32^,^38^ enables the foragers to compensate for the effect of concentration by adjustment of body temperature. One has to keep in mind that the suction pump (cibarium and associated structures) surely has a maximum capacity which cannot be increased further by increasing body (head) temperature. This example shows that consideration of physiological necessities and constraints is important for a proper interpretation^10^.

During limited sucrose flow efficiency was strongly reduced in shade (Fig. 3B) because the bees obviously had to invest considerable energy to keep the thoracic flight muscles at a temperature high enough for immediate take-off (Fig. 2B). With increasing T_a_ the observed reduction of energy turnover (Fig. 2A) allowed just a relatively moderate increase of efficiency in shade (Fig. 3B). The use of solar heat for thermoregulation, on the other hand, allowed for considerable energy savings and this way boosted efficiency (Fig. 3B), which coincides with the report that honeybees prefer flowers in the sun over those in shade^28^. In contrast to our limited flow condition, in a natural situation on flowers also the intake rate may be influenced by the bees to some extent, by choosing more nearby flowers^20^ or by modulating flight speed between flowers. Nevertheless, efficiency optimisation will probably be more important in this case^21^,^34^,^35^. In water foragers, by contrast, maximisation of the (mass) intake rate is more important. There is clear evidence that the level of thermoregulation and energetic expenditure of honeybee foragers depends not only on environmental conditions but also on the bees’ motivational status, which depends on concentration and flow of nectar, the distance from the hive and the demand in the hive^8^,^13^,^16^,^17^,^29^,^31^,^36^,^37^. It follows from this that what is optimal for the individual forager at a certain point of time is variable and not constant.

Honeybee dancing we suggest to be an ’investment-guided’ (‘investing’) behaviour. The additional investment of time and energy during information exchange with colony members^22^,^38^ improves colony intake rate in the first^3^,^23^,^38^ and foraging efficiency in the second place^23^. This is especially effective if the foragers cannot improve the own food intake rate because of limited foraging gains per flower, and if foragers are redirected by the dancers to locations with a better yield^23^,^38^,^39^,^40^.

In conclusion, the data presented here have empirically resolved basic economic mechanisms governing optimisation of honeybee foraging in reaction to environmental parameters. A flexible change between ‘investing’ and ‘economizing’ strategies allows them to balance maximisation of individual and colony intake rate with optimisation of foraging efficiency in their variable environment.

## Materials and Methods

### Experimental procedure

Simultaneous comparison of foraging energetics and thermoregulation in sunshine and shade was done with 22 individually marked honeybees (*Apis mellifera carnica* POLLMANN) originating from 15 colonies in an apiary about 10–20 m away, on 21 days from July to October 2005 between 10:00 and 16:00 hours. They foraged 0.5 M sucrose solution ad libitum or at a flow rate of 15 μl/min from inside a brass measurement chamber of ~7.9 ml inner volume, immersed in a water bath for temperature control (Julabo F33 HT)^29^. The whole setup was placed outside the laboratory in shade or in sunshine (radiation values in Supplementary Fig. S2). The chamber lid could be opened and closed quickly to give the bees fast access to an artificial flower inside^29^.

### CO_2_ production and energetics

The CO_2_ production was measured with a differential infrared gas analyser (DIRGA; URAS 14, ABB) in a flow-through measurement setup in serial mode^29^, operated at a flow rate of 240 ml/min. The loss of measurement gas during chamber opening after the insects’ visits was compensated for by calibrations comparing the washout volumes from the chamber containing certain concentrations of CO_2_ with and without chamber opening^29^. Since in endothermic honey bees we measured a respiratory quotient (RQ) of 1.0073 (SD = 0.0843, N = 25, 7 bees), energy turnover (P) could be calculated directly from CO_2_ production rate (VCO_2_) without the need to convert to O_2_ consumption^41^: P [W] = VCO_2_ [lO_2_ s^−1^] * Caloric equivalent [21.117 kJ lO_2_^−1^ for sucrose feeding bees].

### Thermographic body surface temperature measurement

Observation of behaviour and measurement of body surface temperature were done without behavioural disturbance of the bees with infrared thermography^16^,^29^ (FLIR ThermaCam SC2000 NTS) at a rate of 3–5 Hz through the plastic film covering the measurement chamber lid^29^. The infrared camera was calibrated against a Peltier-driven reference radiator placed close to the insects^29^. The attenuation of the infrared radiation by the plastic film was compensated for by covering part of the reference source head with a stripe of the same film. Together with several layers of corrugated cardboard placed above the measurement setup this also minimised errors resulting from ambient reflections via the film surface.

### Environmental parameters

The ambient air temperature (T_a_) near the foragers (~1 cm) was measured inside the measurement chamber by a thermocouple at the air outlet below the bees. Solar radiation reaching the bees through the plastic film window of the measurement chamber lid was measured by a photoelectric miniature global radiation sensor in a second chamber beside that containing the artificial flower (FLA613GS/Mini spezial; Ahlborn)^29^. Environmental data were recorded by ALMEMO data loggers (2690–8 or 2890–9; Ahlborn).

### Energy gain

The energy gain from sucrose foraging was determined by training the bees to pass a balance (Mettler Toledo) where their landing and take-off weight was measured to the nearest 0.1 mg before and after their visit to the artificial flower. Crop load was calculated from the difference. Energy gain from sugar was determined by using a sucrose solution density of 1.0638 g cm^−3^ for 0.5 mol/l at 20 °C, and a calorific value of 16.8 kJ/g sucrose^23^,^41^.

### Data evaluation and statistics

Respiratory data evaluation was done in Excel (Microsoft) and Origin (OriginLab) software. From the thermographic recordings (dorsal view), the body surface temperature of head, thorax and abdomen was evaluated every 3–5 seconds, using a cuticular emissivity of 0.97 of the honeybee^42^, with ThermaCam Researcher software (FLIR) controlled by a proprietary Excel VBA macro which extracted the stored environmental data (ambient temperature, radiation, etc.) automatically from the logger files at the time of thermographic measurement. Curve fitting and statistics was done with Origin (OriginLab) and Statgraphics (Statpoint Technologies) software.

## Additional Information

**How to cite this article**: Stabentheiner, A. and Kovac, H. Honeybee economics: optimisation of foraging in a variable world. *Sci. Rep.* **6**, 28339; doi: 10.1038/srep28339 (2016).

## Supplementary Material

Supplementary Information
