# Supplementary material for: Honeybee economics: optimisation of foraging in a variable world
Source: Sci Rep. 2016 Jun 20;6:28339. doi: 10.1038/srep28339 (PMC4913264; doi:10.1038/srep28339)
Supplement: Supplementary Information [file srep28339-s1.pdf]

Honeybee economics: optimisation of foraging in a variable world

Anton Stabentheiner[a1], Helmut Kovac[a1]
[a]Institute of Zoology, University of Graz, Universitätsplatz 2, 8010 Graz, Austria.

[1]To whom correspondence may be addressed. E-mail: anton.stabentheiner@uni-graz.at or helmut.kovac@uni-graz.at.

Supplementary Information:

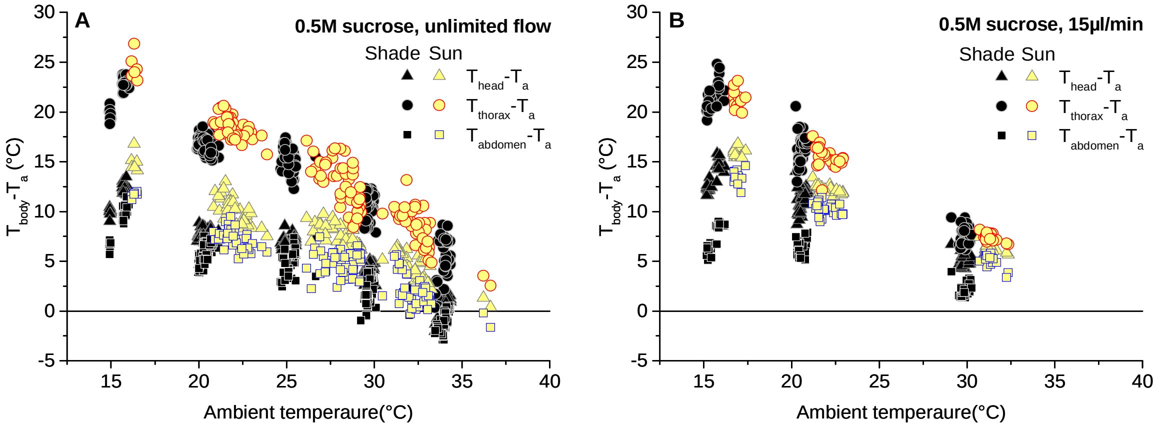

Supplementary Figure S1. Body surface temperature increase over ambient temperature per stay (T_{body}-T_a). Bees (N = 22) foraged 0.5 M sucrose solution from an artificial flower, unlimited (A) and 15 µl/min flow (B). T_a = ambient air temperature.

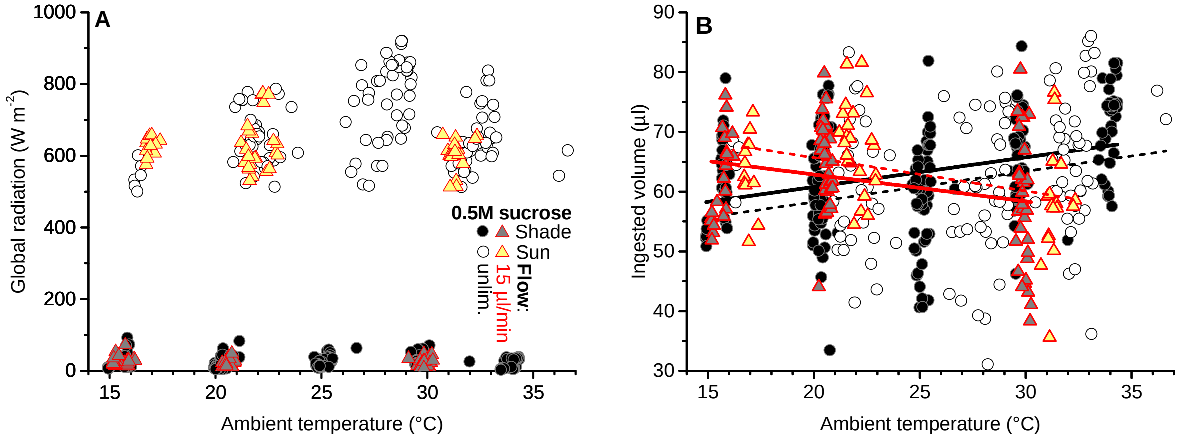

Supplementary Figure S2. Radiation conditions and ingested sucrose volumes per stay. (A) Global radiation at the foraging site (individual stays). Overall means with SD in shade and sunshine, respectively: unlimited sucrose flow, 26.5±15.51 mW (N = 217) and 679.3±107.92 mW (N = 132); 15 µl/min sucrose flow, 22.7±11.75 mW (N = 101) and 619.0±57.48 mW (N = 54). (B) Ingested sucrose volume per stay corrected for sucrose density at 20 °C. Overall means in shade and sunshine, respectively: unlimited sucrose flow, 63.2±9.05 µl (N = 205) and 62.6±11.27 µl (N = 118); 15 µl/min sucrose flow, 61.6±9.14 µl (N = 86) and 63.5±8.86 µl (N = 52). Linear regressions all significant ($P < 0.05$, ANOVA).

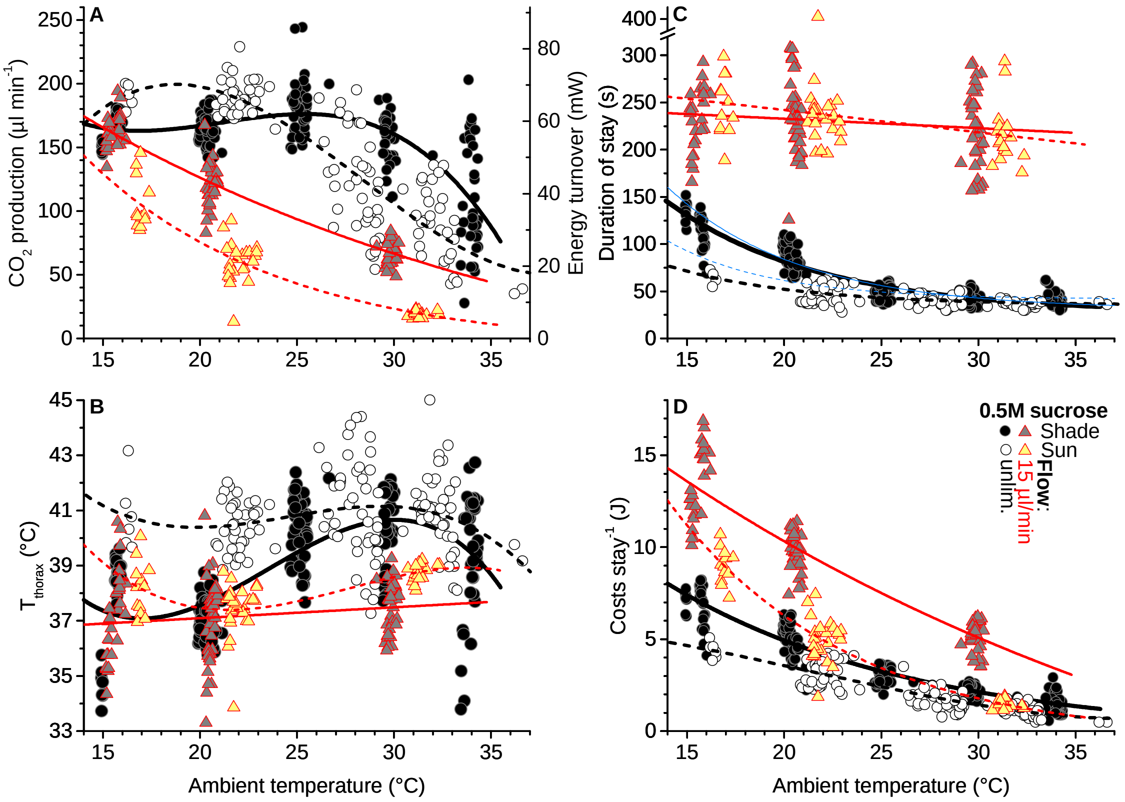

Supplementary Figure S3. Energetics and thermoregulation of sucrose foraging honeybees. (**A**) CO_2 production rate and energy turnover. (**B**) Thorax surface temperature, for head and abdomen see Supplementary Fig. S1. (**C**) Duration of stay. Blue thin lines: 1.5M feeding at unlimited flow, solid = shade, dashed = sun, from[14]. (**D**) Costs per stay. (**A-D**) 22 individuals of *Apis mellifera carnica* foraging 0.5 M sucrose provided in unlimited (unlim.) flow or at a rate of 15 µl/min, in shade (solid lines) or in sunshine (dashed lines), for legend see (D); symbols represent individual stays (visits), for means see Fig. 2; for radiation values see Supplementary Fig. S2, and for regression functions and statistics see Supplementary Table S1.

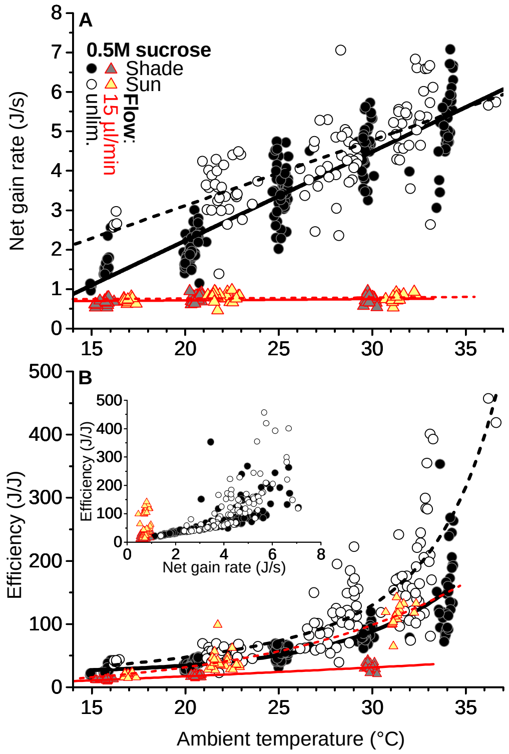

Supplementary Figure S4. Net energy gain rate and foraging efficiency of honeybees. (**A**) Net energy gain rate per stay (gain-costs/second, in J/s). (**B**) Foraging energy efficiency during the stays at the feeder (gain-costs/costs, in J/J); insert, relationship between efficiency and net gain rate. (**A-B**) Symbols represent individual stays (visits), for means see Fig. 3; all relationships significant at P < 0.0001 except 15 µl/min flow conditions in A (n.s.); for regression functions see Supplementary Table S1.

Supplementary Table S1. Constants and statistics for regression functions in Fig. 2 and Fig. 3.

Figure	Sucrose flow	Condi-tion	Function	Parameters					N	R^2
			linear	A	B					
			cubic	A	B	C	D			
			exp. decay	y0	A1	t1	k	tau		
			exp. grow 1	y0	A1	t1	k	tau		
			exp. grow 2	y0	A1	t1	A2	t2		
Fig. 2A	unlimited	shade	cubic	426.111	-42.13843	2.18459	-0.03588		208	0.44536
		sun	cubic	-497.03855	89.01451	-3.54868	0.04172		111	0.73699
	15 µl/min	shade	exp. decay	-64.56389	403.88582	26.66679	0.0375	18.48401	98	0.88415
		sun	exp. decay	-7.23132	608.77333	10.02892	0.09971	6.95152	54	0.82584
Fig. 2B	unlimited	shade	cubic	71.56317	-4.99592	0.23	-0.00326		217	0.37448
		sun	cubic	64.4253	-3.13431	0.13266	-0.0018		131	0.02934
	15 µl/min	shade	linear	36.31168	0.03928				101	0.01715§
		sun	cubic	67.52907	-3.5611	0.13564	-0.00164		54	0.32066
Fig. 2C	unlimited	shade	exp. decay	27.06433	706.85829	7.80444	0.12813	5.40963	208	0.84462
		sun	exp. decay	35.24869	351.89475	6.58622	0.15183	4.56522	112	0.43551
	15 µl/min	shade	linear	252.78991	-0.9964				99	0.01158§
		sun	linear	289.30114	-2.35916				54	0.11347*
Fig. 2D	unlimited	shade	exp. decay	-0.25238	24.59593	12.85045	0.07782	8.90725	217	0.89363
		sun	cubic	5.43304	0.15679	-0.01814	0.000282		131	0.78276
	15 µl/min	shade	exp. decay	-10.21301	37.01831	33.94928	0.02946	23.53185	100	0.84975
		sun	exp. decay	-0.51577	59.77602	9.20269	0.10866	6.37882	54	0.90292
Fig. 3A	unlimited	shade	linear	-2.28221	0.22552				197	0.81911
		sun	linear	-0.19345	0.16548				99	0.49324
	15 µl/min	shade	linear	0.6523	0.00307				83	0.02532§
		sun	linear	0.70787	0.0026				52	0.00111§
Fig. 3B	unlimited	shade	exp. grow 2	21.73391	0.22242	5.98477	0.22242	5.98452	205	0.71473
		sun	exp. grow 2	-1.43384	0.00145	2.98876	11.19994	13.8003	118	0.66894
	15 µl/min	shade	exp. grow 1	-107.03079	100.31452	93.19583	0.01073	64.59843	85	0.84365
		sun	exp. grow 1	-9.37437	5.85209	10.29407	0.09714	7.13531	52	0.8801

Definition of functions: Linear, y = A + B*x. Cubic, $y = A + B*x + C*x^2 + D*x^3$. Exponential (exp.) decay, $y = y0 + A1*e^{(-x/t1)}$; exponential grow 1, $y = y0 + A1*e^{(x/t1)}$; derived parameters: decay rate, k = 1/t1; half life, tau = t1*ln(2). Exponential grow 2, $y = y0 + A1*e^{(x/t1)} + A2*e^{(x/t2)}$. N = number of stays (visits). All regressions significant at P << 0.0001; except *, P < 0.01, and §, P > 0.08 (n.s.); ANOVA, df = N-2 for linear, N-4 for cubic, N-3 for exponential decay and exponential grow 1, and N-5 for exponential grow 2 functions.
